# Are women at high risk for serous gynaecological cancer (SGC) opting for risk-reducing salphingo-oophorectomy motivated by high levels of anxiety and risk perceptions?

**DOI:** 10.1186/1897-4287-10-S2-A5

**Published:** 2012-04-12

**Authors:** B Meiser, MA Price, PN Butow, J Karatas, M Charles, KA Phillips, kConFab Psychosocial and Clinical Follow-Up groups

**Affiliations:** 1 Psychosocial Research Group, Dept of Medical Oncology, Prince of Wales Hospital, Randwick, Australia; 2 Centre for Medical Psychology and Evidence-based Decision-making, and School of Psychology, University of Sydney, Australia; 3 School of Psychology, University of Sydney, Australia; 4 Division of Cancer Medicine, Peter MacCallum Cancer Centre, Australia

## Background

This study assessed sociodemographic, biological and psychosocial determinants of the decision to undertake risk-reducing salpingo-oophorectomy (RRSO).

## Methods

Women participating in the kConFab Clinical Follow-up and Psychosocial studies who were at increased risk for serous gynaecological cancer (SGC) (i.e. BRCA1 or BRCA2 carriers or a family history with at least one first- or second-degree relative with SGC), had no personal history of cancer and had not had an RRSO at the time of kConFab enrolment were included in the analyses. Women who had been informed that they did not carry the BRCA1 or BRCA2 mutation segregating in their family (true negatives) were excluded. Predictor variables were assessed using self-administered questionnaires and interviews at the time of enrolment, and data on RRSO uptake was from the most recent three-yearly follow-up assessment.

## Results

579 women were eligible. Mean age was 43.5 years (range 18 to 74 years). 118 women (20.4%) reported having been tested and knowing that they are mutation positive, while 461 (79.6%) reported not having been tested. 69 women (11.8%) had an RRSO during the follow-up period on average 3 (range 1 month and 8 years) after entry into kConFab. Logistic regression showed that women who had an RRSO were more likely: to be married (OR 2.3, *p*=0.03); to have children (OR 2.6, *p*=0.03); and to know that they are mutation positive (OR 2.9, *p*<0.001), having adjusted for age as a potential confounder (*p*=0.78). None of the psychological variables (breast cancer/SGC-specific anxiety, perceived SGC risk, optimism, social support) were associated with uptake of RRSO, either in the sample as a whole or in interaction with age (<40 years, 40-49, vs 50+).

## Conclusions

These findings are reassuring as they show that women’s decision-making about RRSO is associated with sociodemographic characteristics and women’s knowledge about their carrier status, rather than high levels of anxiety and perceived risk. The limitations of this study include the fact that the psychological variables were assessed in some cases several years prior to RRSO, and these variables might have been different just prior to RRSO.

